# Anesthetic Considerations in Endovascular Stroke Treatment: A Clinical Case Study

**DOI:** 10.7759/cureus.67552

**Published:** 2024-08-23

**Authors:** João Nuno Oliveira, Mariana Santos, João Saraiva, Sofia Xavier, Ângelo Carneiro

**Affiliations:** 1 Neuroradiology Department, Hospital Pedro Hispano, Matosinhos, PRT; 2 Neuroradiology Department, Hospital de Braga, Braga, PRT; 3 Neuroradiology Department, Centro Hospitalar e Universitário do Porto, Porto, PRT

**Keywords:** conscious sedation, general anesthesia, mechanical thrombectomy, angiography, acute ischemic stroke

## Abstract

Acute embolic sequential bilateral occlusion is an extremely rare event and can be difficult to detect during mechanical thrombectomy (MT) under general anesthesia (GA). We describe a male with vascular risk factors who was admitted two hours after the sudden onset of aphasia, right-sided central facial palsy, homonymous hemianopsia, and ipsilateral hemiplegia/hypoesthesia. Thrombolytic intravenous treatment was started, and the patient had an allergic reaction and required intubation. MT was performed under GA, and after the first recanalization, a new contralateral occlusion was detected during the angiographic evaluation, which was also recanalized. Furthermore, we systematically review the literature to identify the prevalence of cases with sequential occlusions described so far and to understand the role of GA in these challenging cases. This allows interventional neuroradiologists to detect such subtle signs, since prompt detection of “de novo” contralateral occlusions with subsequent immediate recanalization is the only way to prevent clinical deterioration in these cases.

## Introduction

Mechanical thrombectomy (MT) is considered the gold standard in the treatment of acute ischemic stroke due to large vessel occlusion when certain eligibility criteria are met [1]. Despite ongoing debate regarding the most appropriate anesthetic approach for the procedure, when general anesthesia (GA) is chosen, the neurological assessment of the patient during and immediately after the procedure is absolutely compromised.

## Case presentation

**Figure 1:**
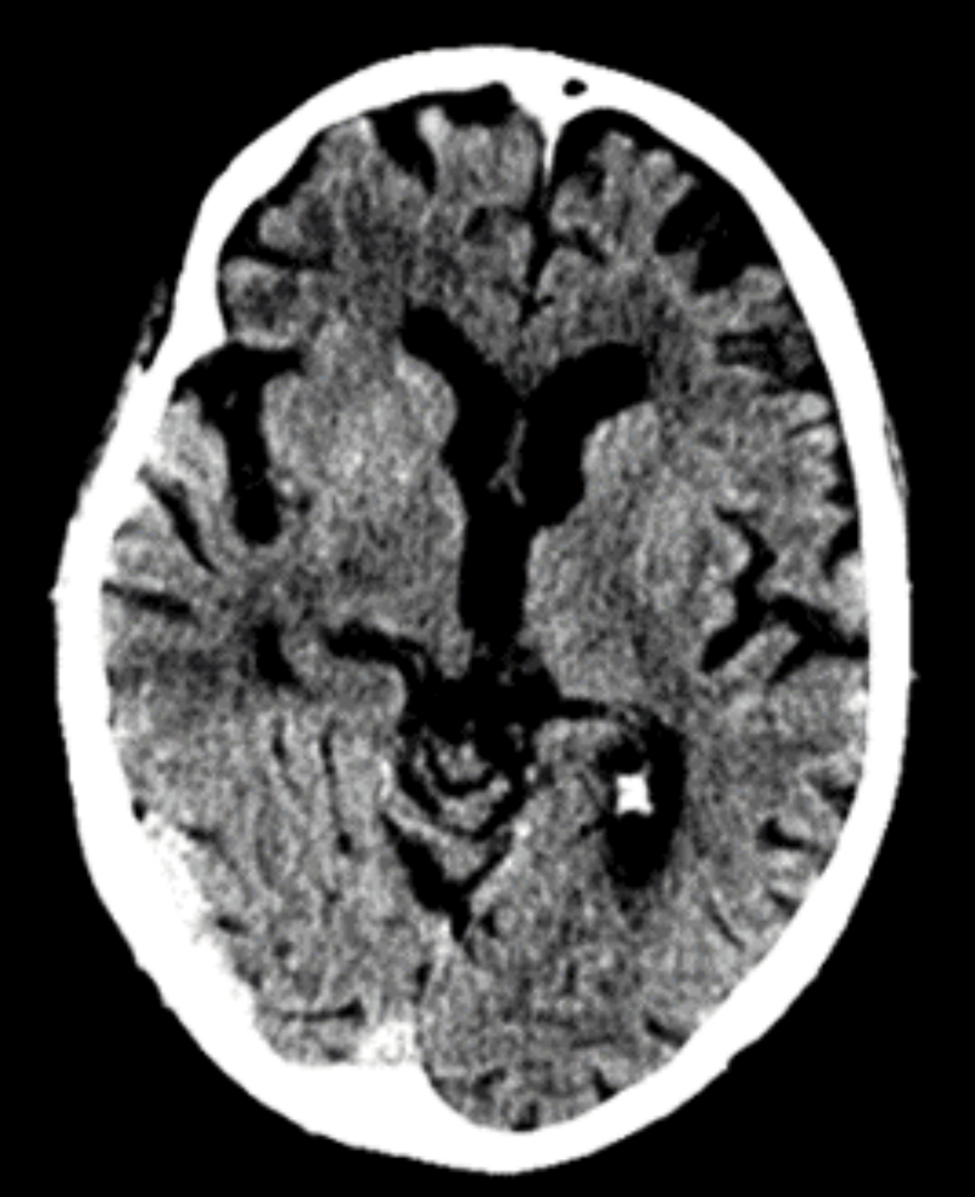
Axial CT brain scan showing an ASPECTS score of 10 with no evidence of acute ischemic or hemorrhagic vascular lesions CT: computed tomography, ASPECTS: Alberta Stroke Programme Early CT Score

**Figure 2:**
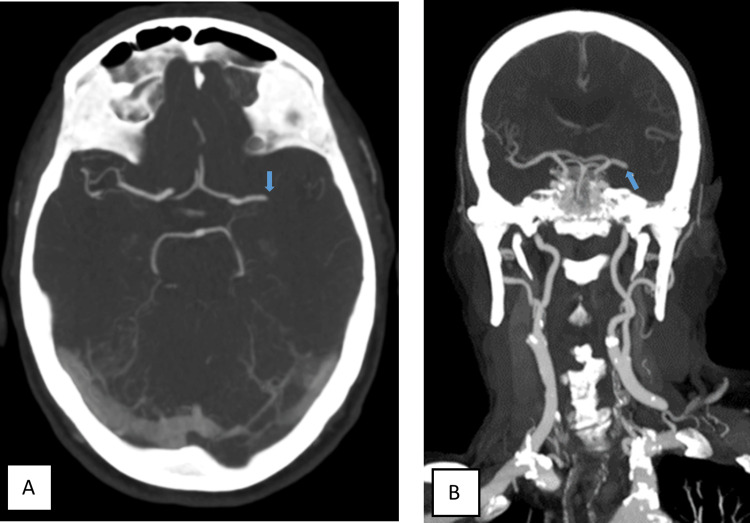
Axial (A) and coronal (B) CT angiography showing the presence of an endoluminal thrombus in the M1 segment of the left middle cerebral artery (blue arrow) CT: computed tomography

**Figure 3:**
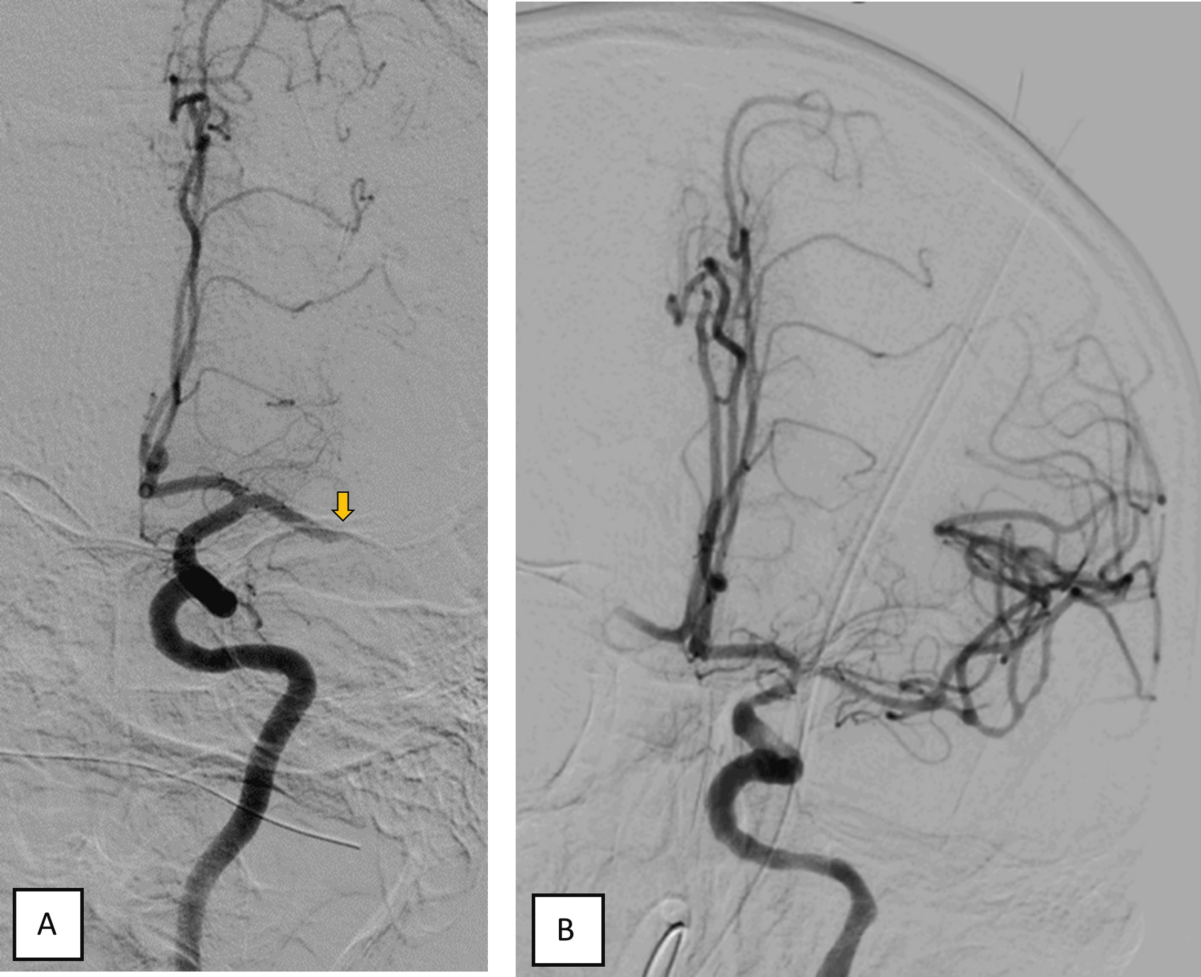
Cerebral angiogram of the left common carotid artery showing the occlusion of the M1 segment of the left middle cerebral artery (A, yellow arrow) and reperfusion of the left middle cerebral artery after MT (B)

An 85-year-old male patient, independent in activities of daily living, was admitted to the emergency department due to aphasia and right-sided hemiplegia with approximately 1.5 hours of evolution. On physical examination, he presented with predominantly motor aphasia, dysarthria, right homonymous hemianopsia, central facial paresis, ipsilateral hemiplegia, and hemihypesthesia (National Institutes of Health Stroke Scale (NIHSS) score of 18). He was hypertensive and had an electrocardiogram trace compatible with atrial fibrillation. The cerebral CT scan did not reveal any acute ischemic or hemorrhagic vascular lesions, showing an Alberta Stroke Programme Early CT Score (ASPECTS) score of 10 (Figure 1). The angiographic study showed the presence of an endoluminal thrombus in the M1 segment of the left medial cerebral artery (MCA) (Figures 2-3).

After initiating intravenous thrombolysis with tenecteplase, the patient developed severe hypertension and vomiting associated with tongue angioedema, leading to intubation for airway protection, and MT was performed under GA. During the MT procedure, reperfusion of the left MCA was achieved with a slight circulatory delay in distal branches (mTICI 2c) (Figure 3).

**Figure 4:**
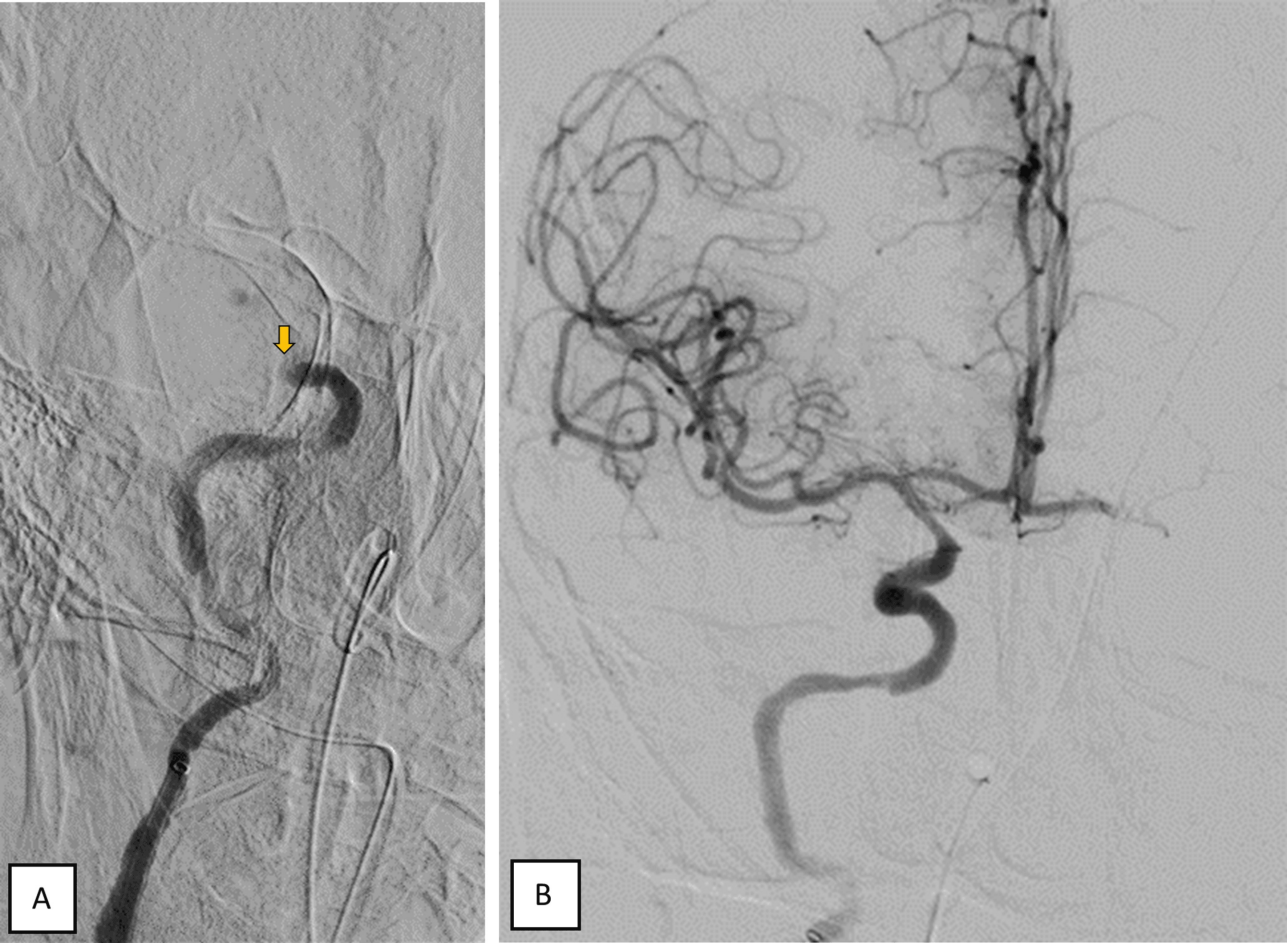
Cerebral angiogram of the right common carotid artery confirmed the occlusion of the top of the ipsilateral internal carotid artery (A, yellow arrow) and complete recanalization after one pass using a stent retriever

During the evaluation of the control angiographic images, contrast retention was identified in the right A1 segment, raising the suspicion of "de novo" contralateral occlusion. Immediate contralateral evaluation confirmed the occlusion of the top of the right internal carotid artery, which was also recanalized with just one pass of a stent retriever, achieving total reperfusion of this territory (mTICI 3) (Figure 4). At discharge, the patient showed significant improvement in neurological deficits, retaining only right hemiparesis grade 4.

## Discussion

Acute embolic sequential bilateral internal carotid artery and/or MCA occlusion is an extremely rare event. The underlying mechanism most likely involves the subsequent scattering of a preexisting intracardiac thrombus [2].

Detecting a sequential intracranial embolism can be quite challenging, especially during MT. Clinical manifestations consist of the sudden appearance of new neurological deficits and, often, deterioration of the level of consciousness. However, during MT, the possibility of clinical monitoring depends on the depth of sedation/anesthesia given to the patient, which raises the debate about the ideal anesthetic management (GA or conscious sedation (CS)) [3]. In CS, we typically administer light to moderate sedatives, enabling patients to follow simple commands and remain still. Physical restraints are avoided unless absolutely necessary. From one perspective, MT under GA seems to allow for higher rates of successful recanalization while maintaining the patient’s immobility and airway protection [4]. Conversely, CS might be associated with a shorter time to recanalization and a lower risk of hemodynamic compromise, and, more importantly, it has the convenience of allowing continuous monitoring of the neurological status [5].

In cases of MT performed under GA, careful reading of the angiographic runs is of the utmost importance and should not be overlooked during the final stages of the procedure. On one hand, this thorough evaluation is mandatory to depict complications such as vessel rupture/perforation, dissections, and distal embolization. On the other hand, it might reveal indirect angiographic signs of previously unnoticed simultaneous bilateral occlusions or sequential contralateral occlusions. One of these angiographic signs consists of leptomeningeal collaterals retrogradely filling an arterial territory; the other is contrast stagnation at a given arterial territory. Whenever the circle of Willis is patent and functional, contrast media should be rapidly washed out by the incoming blood; contrast stagnation indicates a potential occlusion.

When GA is used during MT, clinical detection of contralateral sequential arterial occlusions is most frequently delayed; hence, the depiction of such indirect angiographic signs has the potential to prompt early recanalization and prevent clinical deterioration, as in this case.

## Conclusions

The main disadvantage of performing MT under GA is the inability to provide constant clinical monitoring and the inability to detect potential changes in neurological status during the procedure. In selected cases of patients undergoing MT with this anesthetic technique, systematic evaluation of angiographic images and knowledge of angiographic signs indicating re-embolization or synchronous occlusion of different arterial axes are particularly relevant. This knowledge can prevent late re-intervention and/or multi-territory involvement.
